# Population genetics reveals high connectivity of giant panda populations across human disturbance features in key nature reserve

**DOI:** 10.1002/ece3.4869

**Published:** 2019-01-28

**Authors:** Maiju Qiao, Thomas Connor, Xiaogang Shi, Jie Huang, Yan Huang, Hemin Zhang, Jianghong Ran

**Affiliations:** ^1^ Key Laboratory of Bio‐Resource and Eco‐Environment of Ministry of Education, College of Life Sciences Sichuan University Chengdu China; ^2^ China Conservation and Research Center for the Giant Panda Dujiangyan China; ^3^ Department of Fisheries and Wildlife Michigan State University East Lansing Michigan; ^4^ Wolong National Nature Reserve Wolong China

**Keywords:** conservation genetics, gene flow, giant panda, population connectivity

## Abstract

The giant panda is an example of a species that has faced extensive historical habitat fragmentation, and anthropogenic disturbance and is assumed to be isolated in numerous subpopulations with limited gene flow between them. To investigate the population size, health, and connectivity of pandas in a key habitat area, we noninvasively collected a total of 539 fresh wild giant panda fecal samples for DNA extraction within Wolong Nature Reserve, Sichuan, China. Seven validated tetra‐microsatellite markers were used to analyze each sample, and a total of 142 unique genotypes were identified. Nonspatial and spatial capture–recapture models estimated the population size of the reserve at 164 and 137 individuals (95% confidence intervals 153–175 and 115–163), respectively. Relatively high levels of genetic variation and low levels of inbreeding were estimated, indicating adequate genetic diversity. Surprisingly, no significant genetic boundaries were found within the population despite the national road G350 that bisects the reserve, which is also bordered with patches of development and agricultural land. We attribute this to high rates of migration, with four giant panda road‐crossing events confirmed within a year based on repeated captures of individuals. This likely means that giant panda populations within mountain ranges are better connected than previously thought. Increased development and tourism traffic in the area and throughout the current panda distribution pose a threat of increasing population isolation, however. Maintaining and restoring adequate habitat corridors for dispersal is thus a vital step for preserving the levels of gene flow seen in our analysis and the continued conservation of the giant panda meta‐population in both Wolong and throughout their current range.

## 1 INTRODUCTION

Rare and elusive large‐bodied mammal populations intrinsically occur at low densities (Mumma, Zieminski, Fuller, Mahoney, & Waits, 2015; Taberlet & Bouvet, 1992) and face increasing threats from climate change and anthropogenic influences (Li et al., 2015; Zhu et al., 2013). Managers are frequently tasked with monitoring population sizes, distributions, and connectivity in order to guide management actions. Noninvasive genetic sampling (NGS) is increasingly being used in the conservation and management of threatened animals, as it allows for the estimation of important population parameters such as total size, genetic diversity, and gene flow among populations (Barba et al., 2010; Schregel et al., 2012; Wang et al., 2016; Zhan et al., 2006). Analyses of gene flow grant inference about the functional connectivity of a landscape and have important implications for conservation. Maintaining adequate connectivity both helps to maintain genetic diversity in small subpopulations (Sharma et al., 2012) and allows for recolonization of areas that undergo localized extinctions (Hanski, 1998).

**Figure 1:**
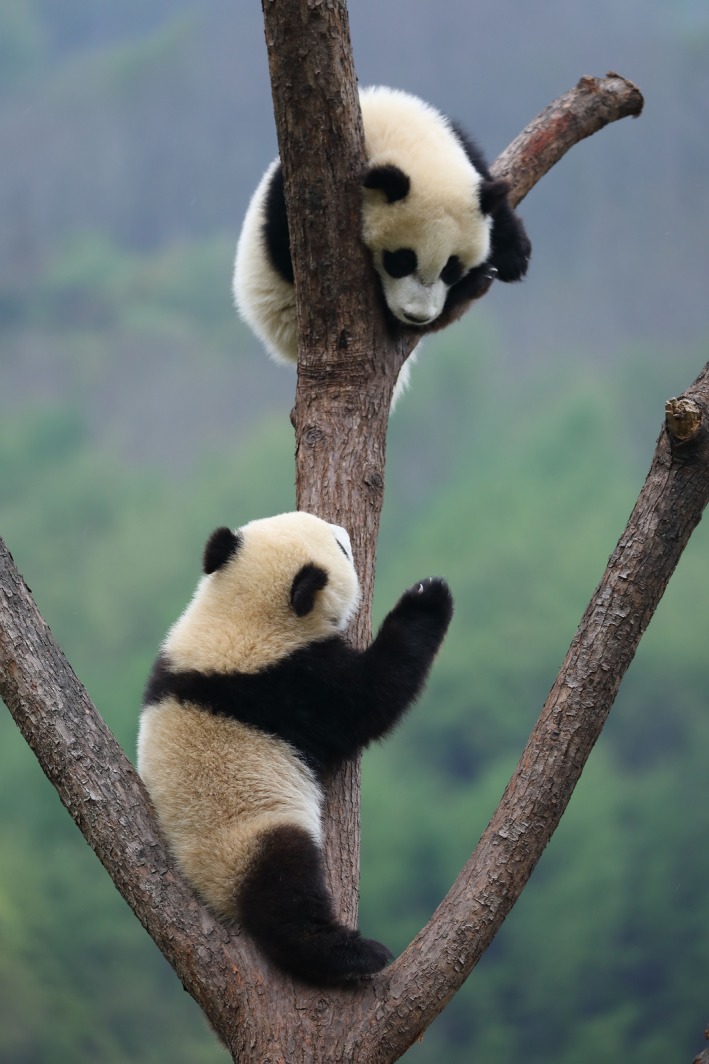
Two captive giant pandas in Wolong. Photo credit to Bo Luo of the China Conservation and Research Center for the Giant Panda.

The giant panda (*Ailuropoda melanoleuca*) (Figure 1) is an example of a species that has faced historical population declines and been the focus of intensive conservation effort through the establishment of protected areas and habitat restoration (Tuanmu et al., 2016). Although there is evidence of recent population recovery which resulted in the reduction of their extinction risk on the IUCN red list (Swaisgood, Wang, & Wei, 2017), pandas still face ongoing increases in habitat fragmentation and subpopulation isolation (Xu et al., 2017; Yang et al., 2017). Currently, their occupancy has been reduced to the eastern edge of the Tibetan plateau in six separate mountain ranges (Schaller, Hu, Pan, & Zhu, 1985). Within these mountain ranges, major rivers, roads, and habitat loss are estimated to further segregate panda populations into 33 subpopulations (State Council Information Office).

Road development in particular has increased substantially across the giant panda range. While roads cover seemingly small proportions of land surface, they affect the environment in various ways, such as through the loss of suitable habitat, animal mortality, acting as barriers to individual movements, and causing landscape fragmentation (Balkenhol & Waits, 2009; Fahrig, 2004; Zhao et al., 2016). These effects can act to increase genetic structure between populations and decrease genetic diversity within populations, which further reduces population viability (Keyghobadi, 2007). This was found to be the case in the giant panda population occupying the Xiangling Mountains, which exhibited genetic differentiation on either side of a major road (Zhu, Zhan, Meng, Zhang, & Wei, 2010; Zhu, Zhang, Gu, & Wei, 2011).

**Figure 2:**
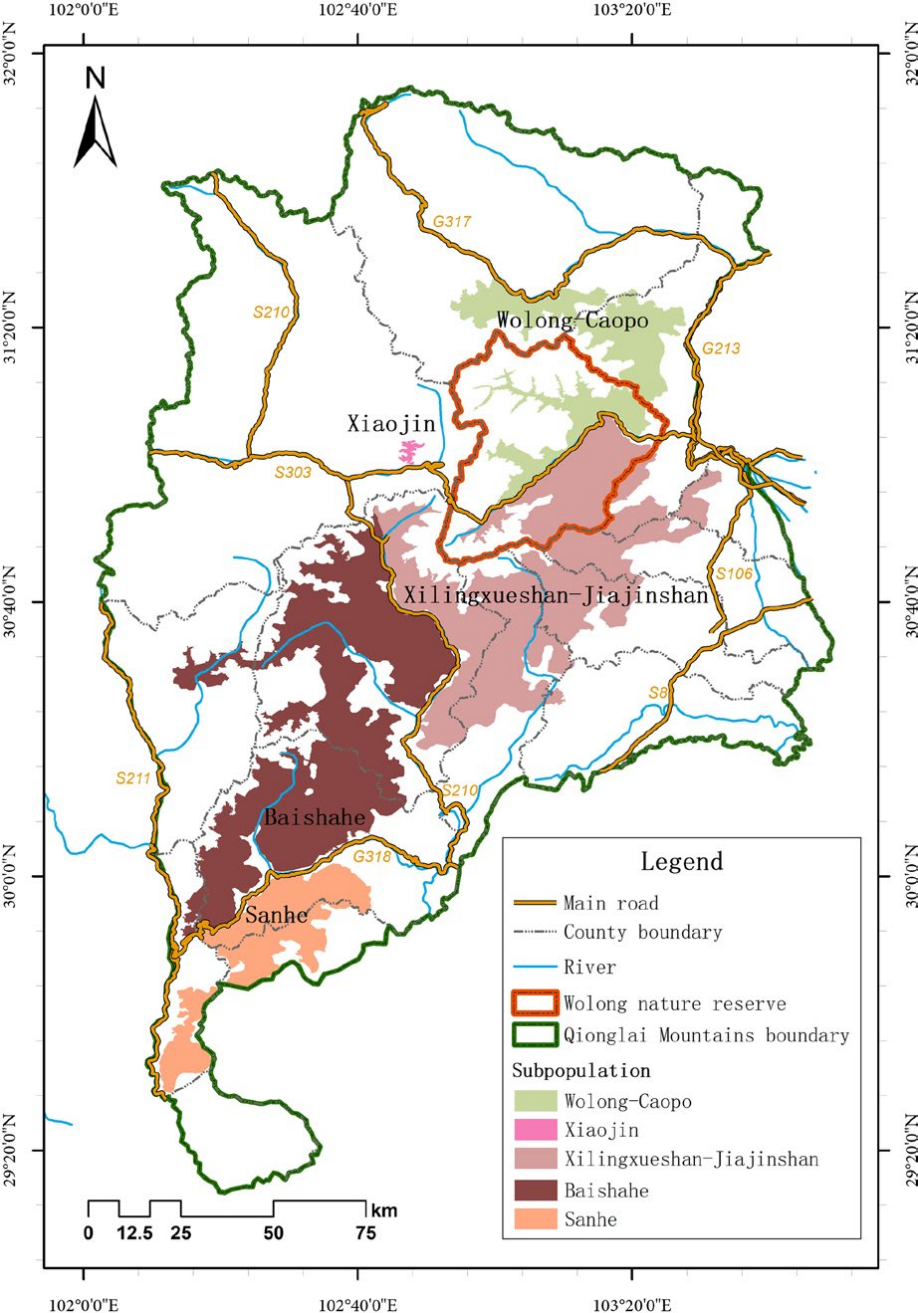
Five giant panda subpopulations purportedly separated by human disturbance events in the Qionglai Mountains.

Of the six mountain ranges occupied by giant pandas, the Qionglai Mountains form the second largest tract of habitat and contain eight nature reserves for giant pandas. Despite this, the Qionglai panda population has been estimated to consist of five subpopulations (Figure 2) (Forestry Department of Sichuan Province, 2015). Two of these subpopulations share a border along a national‐level (G350) road running approximately through the middle of Wolong Nature Reserve, the flagship panda reserve in China and comprising 2,016 km^2^ of rugged mountains situated at the core of panda habitat in the Qionglai Mountains. There are also approximately 5,000 local residents living in three townships situated alongside the national road. The northern Wolong‐Caopo subpopulation and southern Xiling‐Jiajin subpopulation are assumed to be separated by these anthropogenic disturbances (Figure 2) (Forestry Department of Sichuan Province, 2015), but this population substructuring was not based on an analysis of the genetic structure or gene flow. Due to the reserve's rugged terrain and the elusive behavior of pandas, empirical information is lacking about whether the southern and northern subpopulation are connected via effective migration across the road. This would mean that these subpopulations in Wolong act as a single meta‐population, which has implications for their persistence and management. Such empirical evidence of population connectivity is also largely lacking across the giant panda range, as analyses within the occupied mountain ranges have rarely employed genetics methods (Shen et al., 2008; Xu et al., 2006).

The main objective of this study was to thus evaluate the genetic connectivity, through the presence or absence of migration, of the panda subpopulations on either side of the main road through Wolong. We also endeavored to use NGS methods to determine the population size, distribution, and genetic diversity of the panda population in Wolong to better understand their ecology and inform effective conservation. We hypothesized that the population size would be fairly large due to the widespread availability of understory bamboo habitat in the reserve, and that this would translate to high levels of genetic diversity. That said, we also hypothesized that there would be a detectable effect of the road on gene flow. We expect our results to have implications for the conservation of remaining giant panda populations both within Wolong and throughout their range.

## 2 MATERIALS AND METHODS

### 2.1 Study region and sample collection

Our study area consisted of the subalpine regions of Wolong Nature Reserve. This region is situated in a global biodiversity hot spot area and features approximately 905 km^2^ of known and potentially suitable giant panda habitat (Forestry Department of Sichuan Province, 2015). From March 2015 through January 2016, we conducted systematic sampling to collected fecal samples along line transects placed within 520 1.42 × 1.42 km survey grid cells throughout the panda's entire potential distribution area in the reserve.

**Figure 3:**
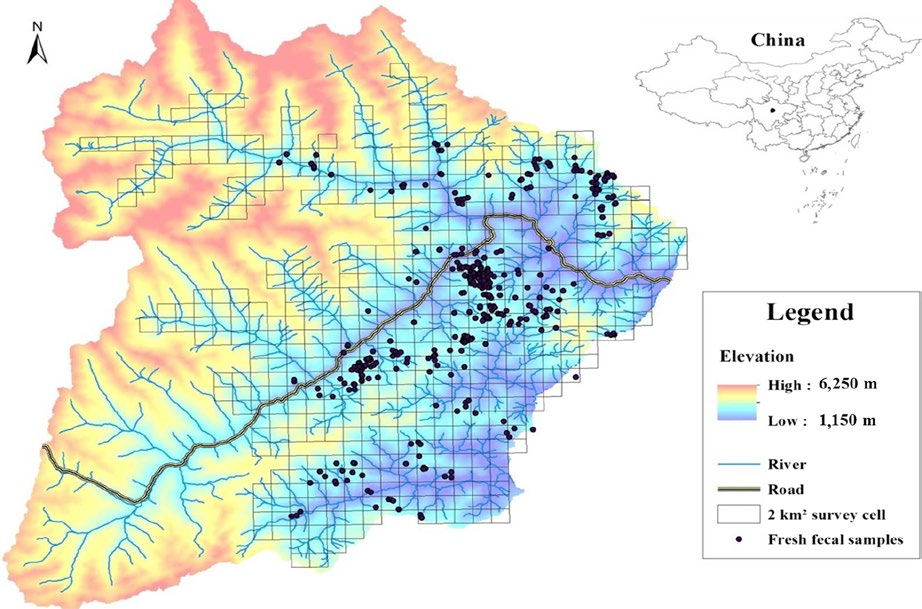
Sampling locations of giant panda feces in Wolong nature reserve, China

A total of 165 trained field workers searched for fresh panda feces, taking a zigzag route in the survey cell in order to collect samples from as many pandas as possible. Most samples were less than two weeks old, based on the condition of the mucosal membrane on the outer layer of the feces. All samples were carefully collected to avoid contamination and preserved in sterile bags or ethanol. All samples were stored at −20°C until DNA was extracted. Each sample was georeferenced using hand‐held GPS units and mapped in ArcGIS‐10. The geographical distribution of sample locations is shown in Figure 3.

### 2.2 DNA extraction and amplification

Total genomic DNA was extracted from fecal samples using QIAamp DNA stool mini kits (Qiagen, Germany), according to the manufacturer's instructions. We used seven tetra‐microsatellite loci to distinguish among individuals. These were as follows: GPL‐60, gpz‐20, GPL‐29, gpz‐6, GPL‐53, GPL‐44, and gpz‐47 (Huang et al., 2015). The probability of identity across these loci in the target population was estimated using GIMLET 1.3.3 (Valière, 2002). PCR amplifications were carried out in 25 μl reaction mixtures comprising approximately 50 ng of template DNA, 2 mm MgCl_2_, 200 μmol of dNTP each, 15 pmol of each primer, 1.0 μg of bovine serum albumin (BSA), and 0.3 units of Hotstart DNA polymerase (Takara). Amplifications were performed using the following PCR procedure: an initial denaturation step for 5 min at 95°C, followed by 35 cycles of 95°C for 45 s, 30 s at locus‐specific annealing temperature and 50 s at 72°C，and a final elongation for 10 min at 72°C. For genotyping, the PCR amplification products were separated by capillary electrophoresis using a denaturing acrylamide gel matrix on an ABI 3730xl Genetic Analyzer. Alleles were detected using Genemapper 3.2 software.

### 2.3 Quality control

Genotyping errors caused by amplification of poor quality DNA from fecal samples such as allelic dropout and false alleles can severely bias estimates of population parameters (Broquet & Petit, 2004; Lampa, Henle, Klenke, Hoehn, & Gruber, 2013). Therefore, we performed control measures to ensure the quality of our genetic data. All fecal samples were amplified at least three times for each marker. A single‐locus genotype was not accepted until our replicates resulted in at least three identical homozygote profiles or two identical heterozygote profiles. These criteria were based on a pilot study, where genotypes obtained from feces versus blood samples were compared (Huang et al., 2015). Huang et al. (2015) concluded that the seven loci used in this study always featured exact matches between blood and fecal samples of *n* = 15 captive pandas, and that results from feces exposed to the natural environment for up to 5 weeks (longer than the estimated 2‐week cutoff of our study) were consistent. As an additional quality control, we used MICRO‐CHECKER to search for loci with large allele dropout and scoring errors caused by stutter peaks (Oosterhout, Hutchinson, Wills, & Shipley, 2004). No evidence of allelic dropout or scoring error due to stuttering was found for any locus. Finally, we used FreeNA to estimate null allele frequencies for each locus (Chapuis & Estoup, 2007). There was an average null allele frequency of <0.04 across the 7 loci.

### 2.4 Estimation of population size

Individual genotypes were identified with the MStools plugin for Microsoft Excel using the following rules: (a) Genotypes from different samples were believed to represent the same individual if all alleles in all loci were identical. (b) If only one allele was found to differ between individuals, DNA was re‐extracted and three more PCR replication was performed. If the allele was still different, we judged the samples as belonging to different individuals. (c) If there were differences of two or more alleles, the samples were accepted as belonging to different individuals.

The noninvasive records of individual genotypes throughout an area can be used to estimate the total population size via capture–mark–recapture methods (Gervasi et al., 2008; Lukacs & Burnham, 2005; Mumma et al., 2015). We used the identification of different individuals through the fecal genetics data to build a CMR model and estimate the giant panda population size in Wolong using the “CAPWIRE” package (Pennell, Stansbury, Waits, & Miller, 2013) in the R programming environment. CAPWIRE performs population size estimation as well as or better than other abundance estimators when the data contain multiple observations of an individual within a session and there are <200 individuals (Miller, Joyce, & Waits, 2005; Mumma et al., 2015). Because our fecal collection efforts focused on all the giant panda's suitable habitat, we inferred that recapture probability was even among all individuals. We thus ran models under the assumption of equal capture (ECM) probabilities in CAPWIRE to estimate the population size. Because our study population was not closed during the study period and there was likely migration across the Northern and Southern borders, we also used the R package “secr” to employ spatially explicit capture–recapture (SECR) methods to estimate a density of pandas per square km across our study area (Efford, 2013). We used the polygon trap format corresponding to the 520 survey cells and grouped the data into 1 session of 30 sampling occasions based on proximity of collection time. We then multiplied the estimated density by the sample area to get an estimate of the number of pandas in Wolong.

### 2.5 Population genetics analysis

The number of alleles (*A*), observed heterozygosity (Ho), expected heterozygosity (He) and polymorphic information content (PIC) were calculated at individual loci and across loci using the software CERVUS 3.0 (Marshall, Slate, Kruuk, & Pemberton, 1998).

A Bayesian clustering method implemented in Structure 2.3.1 (Pritchard et al., 2000) was used to determine the most likely number of genetic clusters. The admixture model was chosen, allele frequencies were assumed correlated, and analysis was conducted with a burn‐in of 100,000 and followed by 1,000,000 MCMC repetitions. Ten independent runs were carried out for each cluster set (*K*), from 1 to 4. The most likely *K* value was determined by evaluating the log likelihood [In *P*(*X*/*K*)] of the posterior probability of the data for each value of *K* (Pritchard et al., 2000). In addition, the △*K* statistic, the second‐order rate of change in the log probability of the data between successive values of *K*, was estimated and used to determine the most likely number of genetic clusters (Evanno, Regnaut, & Goudet, 2005). To cross‐validate the results of STRUCTURE, we also conducted a principal coordinates analysis (PCoA) using GenALEx V6.5 (Peakall & Smouse, 2012). In this analysis, multivariate genetic distances between individuals (Smouse & Peakall, 1999) are decomposed through PCoA to find sources of genetic variation across the population.

For quantifying genetic differentiation between populations, we estimated an *F*_ST_ and its significance value through resampling 10,000 permutations of the genotypes between populations to derive a null distribution using Arlequin 3.5 (Excoffier & Lischer, 2010). Stable and separate populations have high *F*_ST_values, while populations with high migration rates between them tend to have lower *F*_ST_values (Sun & Chang, 2016). Simulations have shown that *F*_ST_ performs better than other indices of population differentiation, as it is more sensitive in detecting population genetic processes when the mutation rate is high relative to the migration rate (Whitlock, 2011). GeneClass v.2.0 was used to detect first‐generation migration of individuals across the road. Specifically, we assigned the two populations on either side different identities before applying Bayesian likelihood‐based test statistics to compute the probability of an individual originating in one of the populations with Monte Carlo resampling of 10,000 simulated individuals at an alpha value of 0.01 (Piry et al., 2004). In this analysis, we estimated the ratio of the likelihood that an individual is of the same population from which it was sampled (*L_home*) divided by the ratio of the highest likelihood of the individual's assignment to any sampled population (*L_max*) to detect migrants.

The Triadic maximum likelihood (TrioML) estimator and the QuellerGt moment estimator, implemented in Coancestry 1.0, were used to calculate the inbreeding coefficient (*f*) for each individual and pairwise relatedness value (*r*) between two individuals, respectively (Wang, 2011). The individual inbreeding coefficient reflects the extent to which their parents are genetically related: *f < *0.125 has been defined as low inbreeding, 0.25 > *f *> 0.125 as moderate, and *f ≥ *0.25 as high inbreeding (Marshall et al., 2002). A smaller negative pairwise relatedness value (*r*) suggests distant kinship, while a larger positive value suggests closer kinship. Offspring of two individuals with a high pairwise relatedness value (*r*) have a high risk of inbreeding deficiencies.

### 2.6 Spatial density pattern

We used the kernel density estimation (KDE) function in ArcGIS 10.0 to quantify the spatial pattern of giant panda density (Bailey & Gatrell, 1995). Previous studies in Wolong have estimated the diameter of giant panda home ranges to fall between 1 and 3 km (Guan et al., 2016; Hu, 2001; Schaller et al., 1985). Supposing that a given giant panda's home range is a circle, the 142 identified panda's GPS locations were used to denote the center of the circle with a radius of 3 km (for pandas with multiple recaptures, we only used the site of the first discovery for this analysis). This circle represented the maximum likely area that a giant panda might utilize. Closer regions to the observed panda locations represent areas of higher probable activity frequencies, which are reflected in the kernel density output. The density map was divided into three tiers (low, medium, and high), indicating different levels of density of inferred space use.

## 3 RESULTS

### 3.1 Sampling and molecular identification

Of the 520 survey cells, we found fresh fecal samples in 140. In total, 539 fresh fecal samples were noninvasively collected for genetic analysis from the entire study area during two sampling sessions in the years 2015–2016 (Figure 3). Successful genotyping of 6 or more microsatellite loci was obtained for 322 samples (with three samples that were successfully genotyped at only 5 loci). The probability of two individuals who were full siblings sharing an identical multi‐locus genotype was 0.00808 based on 6 loci, indicating that this subset of the original 7 loci was enough for accurate individual identification (PIDsib < 0.01) (Waits, Luikart, & Taberlet, 2001). Although using 5 loci resulted in a PIDsib of 0.015, the three samples that were only successfully genotyped at 5 loci were included in the analysis because of large spatial distance between them and other samples (>2 km).

Our molecular analysis identified 142 individual giant pandas in the study area. Identified individuals were observed from 1 to 17 times, with an average of 2.3 samples per individual. 57 (40%) giant pandas were represented by two or more observations, leaving 85 individuals that were only observed once. Our ECM capture–mark–recapture (CMR) model estimated a population size of 164 individuals (95% confidence interval 153–175). The SECR analysis of panda density across the study area estimated 0.13 pandas/km^2^ (95% confidence interval 0.11–0.16), translating to 137 individuals (115–163) in Wolong.

### 3.2 Spatial density pattern

**Figure 4:**
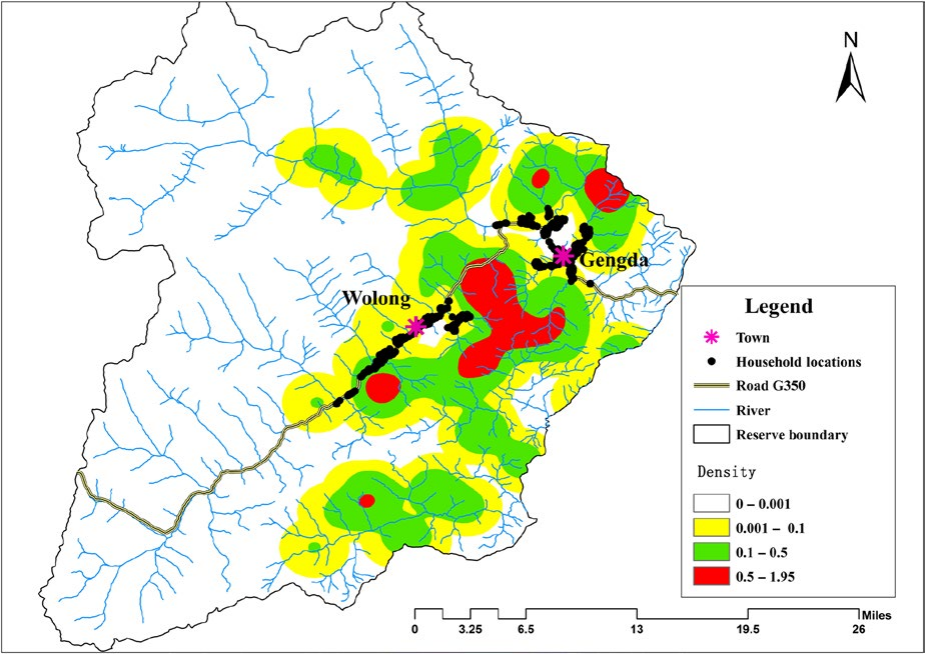
Pattern of giant panda kernel space‐use density in Wolong Nature Reserve (*r* = 3,000 m)

There were four areas with relatively high densities of giant pandas in Wolong: the Tiantaishan, Hetaoping‐Niutoushan, Wuyipeng, and Xihe areas, ordered from north to south (Figure 4). The large home range overlap and spatial proximity of separate areas of activity indicate that in the absence of strong resistance or barriers to movement, the giant pandas in Wolong constitute a relatively continuous population.

### 3.3 Genetic variation and inbreeding

The number of alleles per locus ranged from 5 at locus GPL‐29/GPL‐44 to a maximum of 14 at locus gpz‐20. The mean number of alleles (MNA) was 7.4 per loci for the entire population. The expected heterozygosity (He) ranged from 0.360 to 0.781 (mean 0.633), and the observed heterozygosity (Ho) varied between 0.386 and 0.741 (mean 0.604) across loci. The polymorphism information content (PIC) ranged from 0.336 to 0.742, with an average of 0.586 (Table 1). No significant Hardy–Weinberg disequilibrium was detected after applying the Bonferroni correction (*p* > 0.01).

**Table 1:** Characterization of microsatellite loci for giant pandas in Wolong

Locus	*N*	*A*	Ho	He	PIC	HW
GPL‐29	139	5	0.698	0.685	0.632	NS
gpz‐20	131	14	0.702	0.774	0.748	NS
gpz‐6	142	6	0.690	0.643	0.601	NS
gpz‐47	140	6	0.386	0.360	0.336	NS
GPL‐60	139	7	0.741	0.781	0.742	NS
GPL‐53	135	9	0.578	0.644	0.577	NS
GPL‐44	139	5	0.432	0.548	0.468	NS
Average	–	7.4	0.604	0.633	0.586	–

Note

*A*: number of alleles; He: expected heterozygosity; Ho: observed heterozygosity; HW: significance of Hardy–Weinberg disequilibrium; *N*: number of individuals genotyped; PIC: polymorphic information content.

We found that 62.68% (*n* = 89) of the 142 sampled wild individuals had an estimated inbreeding coefficient of *f* < 0.125, 18.31% (*n* = 26) of the individuals had 0.25 > *f *≥ 0.125%, and 19.01% (*n* = 27) had *f* ≥ 0.25. The average was *f* = 0.135 for the whole population. Genetic relatedness analysis revealed that 68.84% of genotyped pairs had an estimated relatedness value of *r* < 0.125, with an average of ‐0.00013 for the whole population.

### 3.4 Population structure and migration

**Figure 5:**
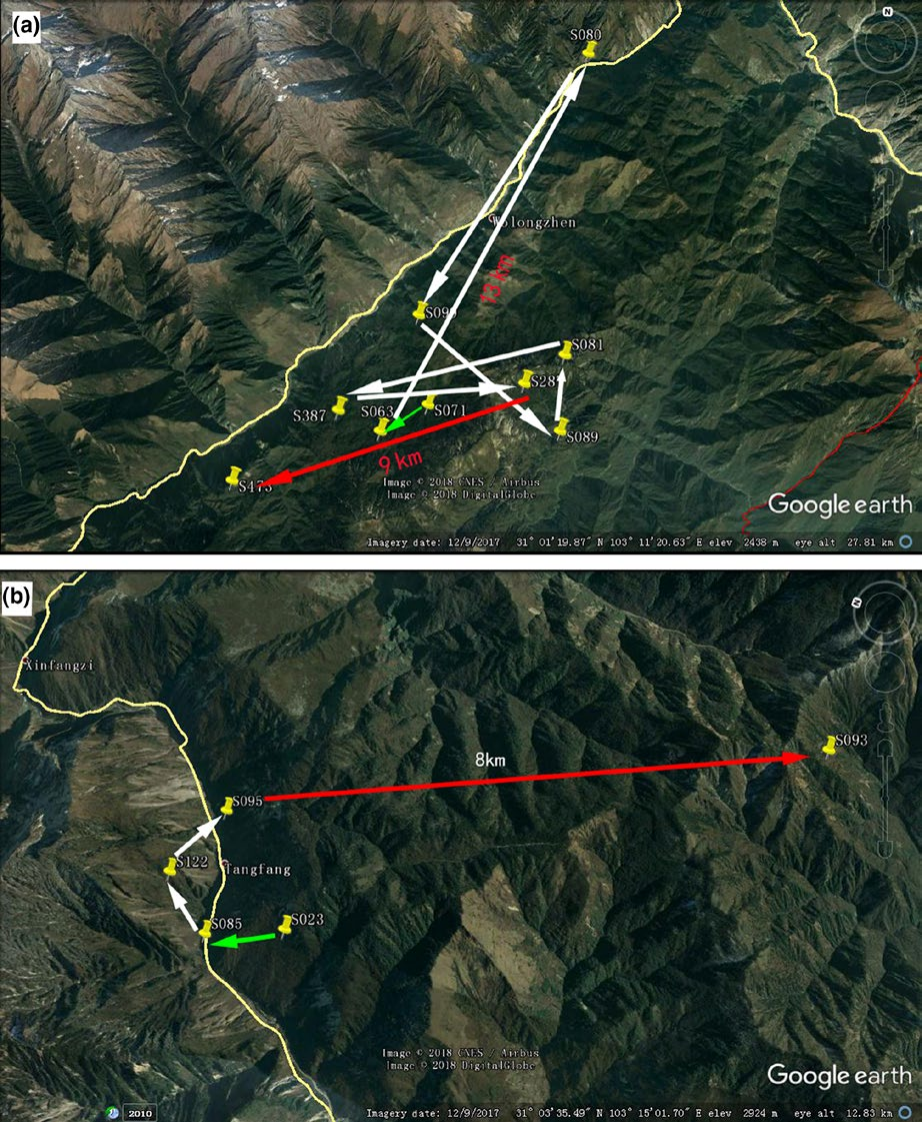
Giant panda WL063 (a) and WL023 (b) crossed the road G350 (yellow line) confirmed by noninvasive individual identification; string of arrows represents the chronological order of the fecal samples

Using the locations of genotyped giant panda scat samples collected from 2015 to 2016, two giant pandas were found to have travelled back and forth across the road (4 total road crossings) in a year (Figure 5). This indicates that pandas were capable of crossing the road G350 during this time period. Moreover, the individual‐based Bayesian likelihood test statistics implemented in GeneClass v.2.0 identified 5 first‐generation migrants across the road.

**Figure 6:**
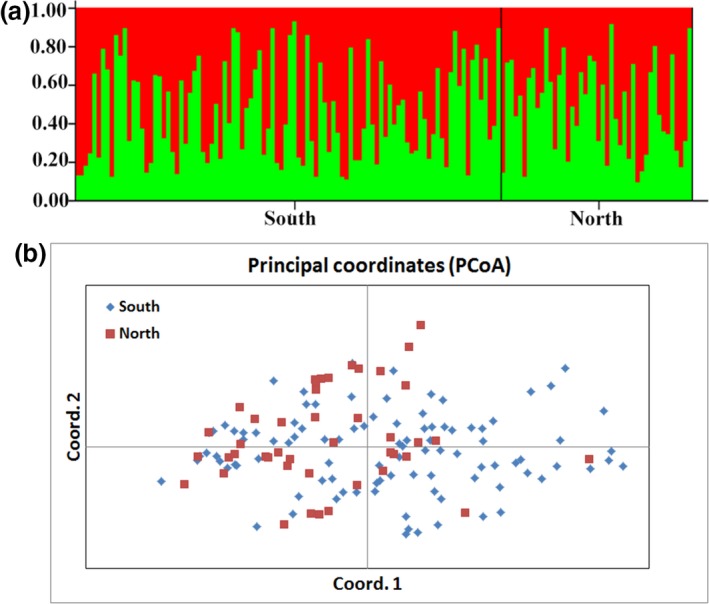
Bayesian clustering plots (*K* = 2) (a) and principal coordinates analysis (PCoA) (b) of the south and north subpopulation separated by road G350 in Wolong

The genetic differences between the southern and northern population around national road G350 were found to be low (*F*st = 0.021 < 0.05, *p* < 0.01), which is equivalent to approximately 12 effective migrants (*N*m) per generation. Bayesian clustering analysis revealed no significant genetic structuring (*K* = 2) between the two populations, with individuals from both south and north of the road forming one genetic cluster (Figure 6). The PCoA based on genetic distances between individuals revealed a similar result, with no clear separation between the same two populations (Figure 6). These results indicate that no significant genetic differentiation has occurred around the G350 national road.

## 4 DISCUSSION

Previous capture/recapture studies of giant pandas that have used genetic markers have resulted in population size estimates that have exceeded those of other methods, with Zhan et al. (2006) estimating nearly double the population size in Wanglang Nature Reserve compare to the 3rd national survey. These methods have been criticized in the past for potential violations of CMR model assumptions, including population closure and genotyping error (Garshelis et al., 2008). Our CMR model estimate of giant panda population size within Wolong was also larger than results from the latest national survey, though not as drastically so (slightly over 50%). The potential for genotyping errors was explicitly addressed in our analysis (see 2.3), but open borders could be a source of bias in the estimation of population size. Because of this, we used SECR methods to model the density of pandas/km^2^ and estimated a total population of 137 individuals. Because SECR estimates density in a spatially explicit manner and thus avoids the assumption of spatial population closure, this is likely a more accurate estimate of the concurrent number of pandas residing in Wolong at a given time. Both this analysis and a simple count of unique genotyped individuals in our study still exceed the estimated population size from the 4th national survey, suggesting Wolong's panda population has not been in the severe decline that national survey results indicate. Increased use of molecular methods and CMR/SECR modeling across a wider area is needed for more accurate monitoring of giant panda population changes over time.

Estimating and evaluating genetic variation is critical for the effective evaluation and management of endangered populations and species (Caniglia, Fabbri, Galaverni, Milanesi, & Randi, 2014; Du et al., 2016; Wang et al., 2016). Populations with higher genetic diversity are often inferred to have greater capacity to adapt to environmental change (Frankham, 2005). Our analysis of genetic variation in the giant panda population in Wolong revealed relatively high levels of genetic diversity with large *MNA*, He, and PIC values. Although the data are not directly comparable because different microsatellite markers were used, comparisons of Wolong populations to other mountain populations suggested that genetic diversity in Wolong ranks relatively high (Supporting information Table S1). On a larger scale, a high level of genetic variation was also confirmed by genomewide SNP analysis of 34 wild pandas' whole genomes, suggesting pandas have large evolutionary potential (Li, Fan, Tian, & Zhu, 2010; Wei et al., 2015; Zhao et al., 2013).

Conservation geneticists emphasize the need to prevent the occurrence of inbreeding in endangered species because it is typically associated with decreased fertility and survival (Deborah & John, 2009; Keller, F., Waller, & Donald, M., 2002; Stevenr et al., 2008). As reflected by our estimated metrics, inbreeding is at a moderate to low level in the Wolong population. Most (68.84%) pairwise individuals had an estimated relatedness value of *r* < 0.125, and most (62.68%) of the individuals had an estimated inbreeding coefficient of *f* < 0.125. This is likely due to the combination of the high rates of migration/gene flow documented in this study and the prevalence for female‐biased natal dispersal supported by collar tracking (Pan et al., 2014; Zhang et al., 2014) and population genetic analysis (Hu et al., 2017; Hu, Zhan, Qi, & Wei, 2010; Zhan et al., 2007). These results are in agreement with previous genomic inbreeding and relatedness metrics calculated using SNP markers from the whole panda genome: Pandas in larger populations like those in the Qionglai Mountains have relatively low levels of inbreeding compared to other mountain ranges (Garbe, Prakapenka, Tan, & Da, 2016).

Our findings that the pandas were able to cross the national road bisecting Wolong, and that this road has not resulted in genetic differentiation between the populations on either side, differ from road effects found in the Xiangling Mountains. Zhu et al. (2010) found that the national road G108 has resulted in a significant degree of genetic differentiation in the giant panda populations there. The smaller effect of road G350 on local panda populations in Wolong (*F*st = 0.021) compared to those of G108 (*F*st = 0.033) could be due to smaller traffic volumes of G350, which was a provincial road S303 before 2017. Generally, wider roads with greater volumes of high‐speed traffic affect wildlife populations more strongly than small, less travelled roads (Clevenger, Chruszcz, & Gunson, 2001; Jaarsma, Langevelde, & Botma, 2006).

Our results are inconsistent with previous studies that have suggested that due to the impact of major roads coupled with the destruction of vegetation nearby, the habitat and panda populations in the Qionglai Mountains have been fragmented into four blocks (Xu et al., 2006). This is directly related to previous assumptions that there has been a lack of gene exchange between the two subpopulations separated by unsuitable habitat and the road G350 in Wolong (Hu, 2001; Loucks et al., 2001; Schaller et al., 1985). These previous efforts to describe panda population substructuring were not based on an analysis of genetic structure and gene flow, however. The relatively high number of effective migrants (*n* = 12) per generation found by our analysis suggests that there have been consistent dispersal events across the road and valley in the recent demographic history of Wolong's giant panda population. Although only 5 first‐generation migrants were detected in our individual‐based analysis of population assignment, this is still enough to produce substantial levels of gene flow and reduce genetic differentiation across the road.

The kernel density map of potential panda activity also supports the conclusion that there is a relatively continuous panda population across Wolong and the central Qionglai Mountains. Although perhaps an overestimation of the actual extent of giant panda space use within home ranges, the output shows that potential home range movements are continuous across the reserve, and notably across the areas of human disturbance. As natal dispersal movements are typically much greater than home range movements in giant pandas (Connor, Hull, & Liu, 2016), the kernel density output represents a conservative estimate, assuming unrestricted movement, of potential population overlap across the reserve. Two areas in particular emerge as likely dispersal corridors—one in between the two human settlements (Wolong and Gengda) and one to the south of Wolong (Figure 4). The sum of our results indicates that the Wolong‐Caopo and Xiling‐Jiajin subpopulations in the Qionglai Mountains are genetically connected with each other via potential dispersal corridors between them. This also has implications for giant panda population connectivity in other areas of the panda range—major roads, even those with further associated habitat disturbances, may not be complete barriers to dispersal. More localized evaluations, even within the scope of larger‐scale research, are thus necessary to understand the effects of anthropogenic disturbance on population connectivity.

Though indicating adequate dispersal and gene flow across the reserve presently, our results do not suggest that conservation action in Wolong or other areas should be lessened. In fact, the maintenance of usable movement corridors across the valley through which the national road G350 runs should be emphasized. Since its successful reconstruction in 2016 after the devastating Wenchuan earthquake in 2008, traffic volumes continue to increase. It is thus likely that successful giant panda dispersal events across the road have declined and will continue to do so, with genetic effects that will manifest in future generations. Corridor preservation and restoration should thus be a priority for managers to maintain the connectivity and levels of gene flow seen in our analysis.

This emphasis on functional connectivity should be more broadly applied to giant panda populations across their current distribution as well, because road construction and increasing traffic volumes have been steadily increasing phenomenon throughout it (Xu et al., 2017). Although our results suggest that population segregation may not be as extensive as suggested in previous analyses (Forestry Department of Sichuan Province, 2015; Xu et al., 2006), full subdivision of presently connected populations is likely an ongoing process. The high levels of genetic diversity frequently seen in giant panda populations (Wei et al., 2015) should thus be seen as a resource to preserve, as well as supplement with reintroduction efforts (Yang et al., 2018). Recent molecular and behavioral investigations suggest that giant pandas rely primarily on adequate dispersal opportunities to avoid inbreeding through sex‐biased dispersal (Hu et al., 2017). This emphasizes the need for habitat connectivity in giant panda conservation. Ultimately, the maintenance of habitat corridors through targeted conservation efforts across the giant panda range will be what continues to ensure stable and healthy wild populations.

Furthermore, additional studies that build on our results should be undertaken. A more detailed analysis of genotypes and their spatial distribution would allow for the reconstruction of wild panda pedigrees and the investigation of small‐scale dispersal patterns. Second, a more comprehensive long‐term noninvasive genetic monitoring of population parameters such as abundance, geographical range shifts, vital rates, and genetic variation would allow for an in‐depth evaluation of population dynamics (Schwartz, Luikart, & Waples, 2007; Waits & Paetkau, 2005). Long‐term genetic monitoring has been successfully used in wild mammals such as brown bear (Barba et al., 2010), coyote (Prugh, Ritland, Arthur, & Krebs, 2005), and wolverine (Bischof, Gregersen, Seth, & H., Ellegren, H. & Flagstad, Ø., 2016; Henrik, Øystein, Cecilia, Malin, & Hans, 2010), but such studies are lacking on giant pandas. NGS offers unique opportunities to acquire the necessary individual identifications from populations that are difficult to observe in order to monitor population dynamics over large timescales. NGS methods have the additional advantage of not disturbing animals to acquire identifications, which is of particular importance for rare and threatened species like the giant panda.

This study thus provides an effective base from which to continue to monitor the giant panda population in Wolong for small‐scale dispersal and population connectivity patterns in the face of environmental changes. Continued study will also allow for the investigation of long‐term population dynamics in order to better understand panda ecology and inform management both within the reserve and throughout the current panda distribution area in order to ensure the continued recovery of one of the world's foremost conservation icons.

## CONFLICT OF INTEREST

None declared.

## AUTHOR CONTRIBUTIONS

Jianghong Ran and Hemin Zhang designed the study. Xiaogang Shi, Thomas Connor, and Yan Huang collected the samples. Maiju Qiao, Jie Huang, and Thomas Connor performed the molecular experiment. Maiju Qiao and Thomas Connor analyzed the data and prepared the figures. Maiju Qiao, Thomas Connor, Jianghong Ran, and Hemin Zhang interpreted the data and wrote the manuscript.

## Data Availability

The microsatellite data available from the Dryad Digital Repository: https://doi.org/10.5061/dryad.hf03sm4.
