# Supplementary material for: Population genetics reveals high connectivity of giant panda populations across human disturbance features in key nature reserve
Source: Ecol Evol. 2019 Jan 28;9(4):1809–19. doi: 10.1002/ece3.4869 (PMC6392360; doi:10.1002/ece3.4869)
Supplement: Supplementary file 1 [file ECE3-9-1809-s001.docx]

Table S1. Summary of genetic diversity parameters of microsatellite data of several giant panda populations

| Population | Mountain | Sample  Size | MNA | *Ho* | *He* | Researcher |
| --- | --- | --- | --- | --- | --- | --- |
| 5 populations | Qin | 32 | 3.5 | 0.525 | 0.486 | (Zhang et al. 2007) |
| Wanglang | MS | 66 | 5.4 | 0.625 | 0.609 | (Zhan et al. 2006) |
| Tangjiahe | MS | 42 | 8.9 | 0.686 | 0.703 | (Yang et al. 2011) |
| 5 populations | QL | 40 | 5.3 | 0.595 | 0.610 | (Zhang et al. 2007) |
| Wolong | QL | 142 | 7.4 | 0.604 | 0.633 | this study |
| Daxiangling | DXL | 21 | 4.7 | 0.660 | 0.634 | (Zhu et al. 2011) |
| Xiaoxiangling | XXL | 32 | 4.6 | 0.704 | 0.656 | (Zhu et al. 2011) |
| Liangshan | LS | 52 | 4.0 | 0.683 | 0.592 | (Hu et al. 2010) |

Note—MNA, mean number of allele per locus; QIN, Qinling Mountains; MS, Minshan Mountains; QIO, Qionglai Mountainss; DXL, Daxiangling Mountains; XXL, Xiaoxiangling Mountains; LS, Liangshan Mountains.

References:

Hu, Y., Qi, D., Wang, H. & Wei, F. 2010. Genetic evidence of recent population contraction in the southernmost population of giant pandas. *Genetica,* 138, 1297-1306.

Yang, J., Zhang, Z., Shen, F., Yang, X., Zhang, L., Chen, L., Zhang, W., Zhu, Q. & Hou, R. 2011. Microsatellite variability reveals high genetic diversity and low genetic differentiation in a critical giant panda population. *Current Zoology,* 57**,** 717-724.

Zhang, B., Li, M., Zhang, Z., Goossens, B., Zhu, L., Zhang, S., Hu, J., Bruford, M. W. & WEI, F. 2007. Genetic viability and population history of the giant panda, putting an end to the "evolutionary dead end"? *Molecular Biology and Evolution,* 24**,** 1801-1810.

Zhan, X., Li, M., Zhang, Z.*,* Goossens, B., Chen, Y., Wang, H., Bruford, M. W. & Wei, F. 2006. Molecular censusing doubles giant panda population estimate in a key nature reserve. *Current Biology,* 16, R451-452.

Zhu, L., Zhang, S., Gu, X. & Wei, F. 2011. Significant genetic boundaries and spatial dynamics of giant pandas occupying fragmented habitat across southwest China. *Molecular Ecology,* 20, 1122-1132.
